# Value of estimated pulse wave velocity to identify left ventricular hypertrophy prevalence: insights from a general population

**DOI:** 10.1186/s12872-022-02541-9

**Published:** 2022-04-08

**Authors:** Yang Liu, Kai Xu, Shaohui Wu, Mu Qin, Xu Liu

**Affiliations:** Department of Cardiology, Shanghai Chest Hospital, Shanghai Jiao Tong University, 241 West Huaihai Road, Shanghai, China

**Keywords:** Aortic stiffness, Estimated pulse wave velocity, Left ventricular hypertrophy

## Abstract

**Background:**

Aortic stiffness shares a similar profile of risk factors with left ventricular hypertrophy (LVH) and can also lead to LVH by itself. Published data have demonstrated the correlation between aortic stiffness and LVH. Recent data have revealed estimated pulse wave velocity (ePWV) to be a simple and cost-effective marker of the severity of aortic stiffness. Our analysis aimed to explore the association between ePWV and LVH prevalence, and to investigate the incremental value of ePWV for the identification of LVH prevalence.

**Methods:**

The present analysis based on a cross-sectional survey which included 11,597 participants from rural areas of southeastern China between Sep 2020 and Feb 2021. ePWV was formulated based on mean blood pressure and age according to a published algorithm.

**Results:**

The prevalence of LVH was 14.56%. With the adjustment of age, sex, education, income and physical activity level, current drinking and smoking status, BMI, waist circumference, serum creatinine, total cholesterol, high density cholesterol, mean blood pressure, fasting plasma glucose, anti-hypertensive therapy, anti-diabetic therapy, lipid-lowering therapy, and cardiovascular disease history, every standard deviation increment of ePWV associated with a 2.993 times risk of LVH prevalence. When dividing ePWV into quartiles, the top quartile had a 4.520 times risk of LVH prevalence when compared with the bottom quartile. Furthermore, smooth spline analysis displayed that the association was linear in the whole range of ePWV (*p* for non-linearity = 0.073). Additionally, subgroup analysis revealed the association was robust to sex, obesity and diabetes, and younger people and hypertensive population were more vulnerable to the increase of ePWV than their corresponding counterparts. Finally, ROC analysis showed a significant advancement when introducing ePWV into established risk factors (0.787 vs. 0.810, *p* for comparison < 0.001), and reclassification analysis also confirmed significant improvement from ePWV to identify LVH prevalence (category-free net reclassification analysis = 0.421, *p* < 0.001; integrated discrimination index = 0.023, *p* < 0.001).

**Conclusion:**

Our analysis demonstrated a linear association between ePWV and LVH prevalence. Furthermore, our results suggest younger people and hypertensive population are more likely to have LVH prevalence with the increase of ePWV. More importantly, our findings implicate the incremental value of ePWV to optimize the identification of LVH prevalence in a general Chinese population.

**Supplementary Information:**

The online version contains supplementary material available at 10.1186/s12872-022-02541-9.

## Introduction

Left ventricular hypertrophy (LVH), a target organ dysfunction resulted by several cardiovascular risk factors, is regarded as a vital indicator of sub-clinical cardiovascular diseases (CVD), and has been demonstrated as the pathophysiological basis of several adverse cardiovascular events, including morbidity and mortality from coronary heart disease, congestive heart failure, stroke, and intermittent claudication [1–3]. Evidence about the prevalence of LVH in general population is scarce, but previous studies have identified high prevalence of LVH in patients with common cardiovascular risk factors like hypertension and diabetes [4–6]. Due to this grim situation, an approach to optimize and simplify the identification of LVH, especially in the primary care condition where cardiac ultrasonography is rarely equipped, is fundamental to relieve the cardiovascular health burden.

Aortic stiffness, a functional and structural marker of cumulative exposure to cardiovascular risk factors, is considered as the arterial memory to cardiovascular injuries [7]. Major risk factors of LVH are also contributors for aortic stiffness, including hypertension, diabetes, and renal disease [8, 9]. Therefore, the level of aortic stiffness may act as a surrogate of the cumulative damage of the left ventricle, and thereby represent the severity of LVH. And current expert consensus has recommended carotid-femoral pulse wave velocity (cfPWV) as the gold-standard of aortic stiffness [10]. Accordingly, previous studies have demonstrated the significant associations between cfPWV or branchial-ankle PWV (baPWV) and LVH prevalence [11–13]. However, although the cfPWV or baPWV have standardized measurement procedures [14], their measurement requires specialized and expensive devices which are rarely equipped in clinical practice, especially in primary care settings [15]. Therefore, daily measurement of cfPWV or baPWV to monitor the severity of aortic stiffness seems impossible. In this regard, there is a need to simplify the technology and research into affordable approach to measure or estimate aortic stiffness.

To address the above problem, previous investigators have formulated the estimated pulse wave velocity (ePWV) to estimate the level of aortic stiffness through an algorithm including age and mean blood pressure (MBP) [16]. The ePWV has displayed a close correlation with the measured cfPWV [16]. Therefore, daily measurement of ePWV can be employed to monitor the level of aortic stiffness. Furthermore, previous investigations have also demonstrated the predictive impact of ePWV for stroke, myocardial infarction, and cardiovascular mortality among several western populations [17–19]. However, limited data have evaluated the association between ePWV and LVH prevalence in the general Chinese population, and no study has investigated the impact of ePWV in the identification of LVH prevalence. Accordingly, the current work aims to analyze the association between ePWV and the LVH prevalence, and to further investigate the incremental value of ePWV to optimize the identification of LVH prevalence in a general Chinese population.

## Methods

### Study population

**Fig. 1:**
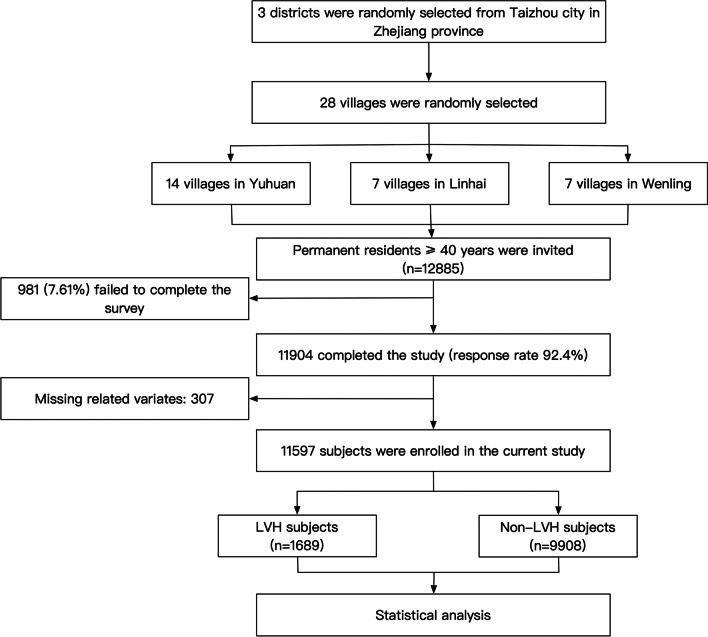
Flow chart of the enrolling process

Our current work derived from a cross-sectional survey conducted in the rural areas of southeastern China. The survey started at September 2020 and ended in February 2021. A multi-stage, geologically stratified and clustered random sampling approach was adopted to improve the representativity of the included participants. The survey randomly selected 28 villages from 3 districts of Taizhou city in Zhejiang province. Eligible subjects were permanent residents aged ≥ 40 years old (n = 12,885). Excluding criteria included pregnancy, cancer, and mental disorders. A total of 11,904 subjects completed the survey. In our present work, additional 307 subjects were excluded because of the censored data of co-variates, and finally enrolled 11,597 participants into statistical analysis (Fig. 1). The central ethics committee of Yuhuan second people's hospital (ethical clearance number: 2020031) and Shanghai Chest Hospital Affiliated to Shanghai Jiao Tong University approved the study protocol of the survey, and the survey was performed according to the principles of the Declaration of Helsinki. A written informed consent was provided by every enrolled participant, their relatives provided the written informed consent if the participants were disabled.

### Data collection and measurement

A crew of epidemiological specialists, cardiologists, and neurologists was engaged to conduct the data collection work. All the members underwent a specialized epidemiological course and passed a final exam before the beginning of the data collection. The clinics in the villages were built for primary medical care under the certification of the local health commission. The clinics were equipped with large and warm rooms which are suitable for performing epidemiological survey. Furthermore, the survey adopted a double-entry approach to improve data quality, which ensured the authenticity and accuracy of the data.

During a single clinical visit, a standard questionnaire was conducted. The questionnaire collected demographic data, including age, sex, socioeconomic status (education and income level) and lifestyle information (physical activity, smoking and drinking habits). Physical activity was documented and classified according to subjects’ answer towards our questionnaire. Average moderate activity (moderate sweating at cool temperature) for less than 2 h per day was defined as low physical activity; average moderate activity for 2–5 h per day was classified as moderate physical activity; average moderate activity for more than 5 h per day was determined as high physical activity. Cardiovascular disease (CVD) history was recorded according to the subjects’ self-reports.

When measuring anthropometric parameters, subjects were asked to wear light clothes without shoes. The standard weight was recorded by a calibrated electric scale to the nearest 0.1 kg. Subjects held in a standing position when a calibrated stadiometer recorded their standard height to the nearest 0.1 cm. Waist circumference (WC) was measured at the horizontal level 1 cm above the umbilicus when the subjects held in standing position.

Calibrated electronic sphygmomanometers (HEM 907, Omron, Kyoto, Japan) were used for measurement of blood pressure. Measurement was performed in the quiet, large, and warm room of the clinics. Exercise, caffeine intake and smoking were forbidden for at least 30 min before any blood pressure measurement. 3 consecutive measurements were recorded for each participant with a 2-min interval between every 2 measurements, and the mean value of the 3 measurements was used for data analysis.

2-Dementional echocardiographers (Vivid, GE Healthcare, United States) were used to capture echocardiograms. The transthoracic echocardiogram was conducted when patients held in supine position. 3 cardiologists who specialized in echocardiography was employed to conduct, read and analyze the echocardiography. Orientation of planes for echocardiographic imaging were performed according to published procedures [20]. Linear internal measurements of the LV were acquired in the parasternal long-axis view carefully obtained perpendicular to the LV long axis, and measured at the level of the mitral valve leaflet tips. Electronic calipers were positioned on the interface between myocardial wall and cavity and the interface between wall and pericardium. Left ventricular end-diastolic internal diameter (LVEDd), interventricular septal thickness (IVST), and posterior wall thickness (PWT) were calculated according to the guidelines of the American Society of Echocardiography [21]. LVEDd was defined as the maximal diameter of left ventricular chamber at the parasternal long-axis view and mitral valve leaflet tips level; IVST was defined as the maximal thickness of interventricular septum at the same view and level; PWT was determined as the maximal thickness of left ventricular posterior wall at the same view and level.

After more than 8 h of fasting, fasting blood samples were collected from every subject via venous puncture at left or right cephalic vein. The blood samples were stored in EDTA vacutainer tubes (Becton, Dickinson and Co., Franklin Lakes, NJ, USA). Centrifugation was performed at the epidemiological site immediately to isolate serum from the whole blood. And then the samples were stored at − 20 Celsius degree. Subsequently, the samples were transported to a certified laboratory at Yuhuan second people's hospital for quantitative analysis. A HITACHI auto-analyzer (H700, HITACHI, Tokyo, Japan) was used to analyze the quantity of serum creatine (Scr), fasting plasma glucose (FPG), triglycerides (TG), total cholesterol (TC), low-density lipoprotein cholesterol (LDL-c), and high-density lipoprotein cholesterol (HDL-c). Additionally, 8 percentage of the serum samples were randomly selected and re-tested in the Clinical Laboratory of a 3^rd^ party institute to enhance the data quality of the laboratory test.

### Definitions

Anti-hypertensive therapy was defined as use of anti-hypertensive drugs in the past 2 weeks. Hypertension was diagnosed as mean systolic blood pressure (SBP) ≥ 140 mmHg and/or mean diastolic blood pressure (DBP) ≥ 90 mmHg, and / or self-reported anti-hypertensive therapy [22]. Anti-diabetic therapy referred to use of glucose-lowering drugs in the past 2 weeks. Diabetes was defined as FPG ≥ 7 mmol/L and/or self-reported anti-diabetic therapy [23]. Lipid-lowering therapy was determined as use of lipid-lowering drugs in the past 2 weeks. Mean blood pressure (MBP) was calculated as DBP + 0.4 (SBP-DBP). ePWV was formulated as ePWV = 9.587 − 0.402 × age + 4.560 × 10^−3^ × age^2^ − 2.621 × 10^−5^ × age^2^ × MBP + 3.176 × 10^−3^ × age × MBP-1.832 × 10^−2^ × MBP [16]. Left ventricular mass (LVM) was calculated based on a necropsy validated formula: LVM = 0.8 × {1.04 × [(IVST + PWT + LVEDd)^3^ − LVEDd^3^]} + 0.6 g [24]. Left ventricular mass index (LVMI) was defined as LVM/(height^2.7^) [25]. LVH was determined as LVMI > 49.2 g/m^2.7^ for males and > 46.7 g/m^2.7^ for females [25]. Relative wall thickness (RWT) was defined as (2 * PWT)/LVEDd [21]. LV geometry was grouped into 4 classes: normal geometry (no LVH & RWT ≤ 0.42), concentric remodeling (no LVH & RWT > 0.42), eccentric hypertrophy (LVH & RWT ≤ 0.42), concentric hypertrophy (LVH & RWT > 0.42).

### Statistical analysis

In statistical analysis, LVH group was defined as group 1, non-LVH group was named as group 2. Continuous variates were showed as mean values (standard deviation, SD) or median (quartile 1–quartile 3) according to the distributions. Categorical variables were displayed as frequency (percentage). Difference between continuous variates with normal distribution was evaluated by student’s t test, and difference between continuous variates with skewed distribution was tested by Mann–Whitney test. Comparison of categorical variates between groups was conducted through Chi-square test, disparity of ordinal categorical variates between subjects with LVH or without LVH was revealed by Rank-Sum test. In our work, we use normalized ePWV to replace unnormalized ePWV to act as a continuous variate because each unit change of normalized ePWV equal to 1 SD change of unnormalized ePWV. Therefore, readers can have a better understanding of intensity of the association between ePWV and LVH prevalence because 4 SD change of ePWV can cover over 95% of the total range of ePWV. The normalization process followed the formula: normalized ePWV = (ePWV − mean ePWV)/SD of ePWV. The independent association between ePWV and LVH prevalence was evaluated by multivariate logistic regression with adjustment of co-variates. The results were displayed as odds ratios (ORs) and 95% confidence intervals (95% CI). Moreover, our work employed a generalized additive model with a spline smoothing function and logarithmic likelihood ratio test to evaluate whether the association between ePWV and LVH prevalence was linear in the whole range of ePWV. Finally, our study employed receiver operating characteristic (ROC) curve, category-free net reclassification index (NRI) and integrated discrimination index (IDI) to investigate the value of ePWV in optimizing the identification of LVH prevalence. All the statistical analysis was conducted through SPSS 26.0 software (IBM corp), statistical software packages R (http://www.R-project.org, The R Foundation) and EmpowerStats (http://www.empowerstats.com, X&Y Solutions, Inc., Boston, MA). Statistical significance was defined as a two-tailed P value less than 0.05.

## Results

Data characteristics of the 11,597 participants were summarized in Table 1. The prevalence of LVH was 14.56%. As for the demographic data, group 1 had significantly higher age, lower education, income and activity level, and lower percentage of male than the group 2. Current smoking and drinking status were significantly lower in group 1. Group 1 had lower height, higher weight and consequently higher BMI and WC level than the group 2. Blood pressure parameters were also significantly worse in the group 1. Regarding the laboratory data, Scr, FPG, TC, TG, LDL-c were higher in group 1 while HDL-c was lower in the group 1. More percentage of subjects in the group 1 had anti-hypertensive, anti-diabetic and lipid-lowering therapy, and therefore group 1 had higher percentage of hypertension and diabetes than the group 2. Subjects with CVD history were also significantly more in the group 1 when compared by percentage. Parameters of left ventricular geometry were worse in the group 1 than group 2. A cross-table was employed to evaluate whether physical activity will influence LV geometry (Additional file 1: Table S1), the results demonstrated that the influence from physical activity was marginal. Finally, the ePWV value was significantly higher in group 1 than that in group 2.

**Table 1:** Characteristics of subjects divided by the presence of LVH

Variables	Total (n = 11,597)	LVH (Group 1, n = 1689)	non-LVH (Group 2, n = 9908)	*p* Value*
Age (years)	53.83 ± 10.57	59.50 ± 10.29	52.86 ± 10.31	< 0.001
Male (%)	5367 (46.28%)	730 (43.22%)	4637 (46.80%)	0.006
*Education level (%)*				
Primary school or below	5778 (49.82%)	1086 (64.30%)	4692 (47.36%)	< 0.001
middle school	4722 (40.72%)	500 (29.60%)	4222 (42.61%)	
high school or above	1097 (9.46%)	103 (6.10%)	994 (10.03%)	
*Income (CNY) (%)*				< 0.001
≤ 5000	1437 (12.39%)	303 (17.94%)	1134 (11.45%)	
5000–20,000	6328 (54.57%)	947 (56.07%)	5381 (54.31%)	
> 20,000	3832 (33.04%)	439 (25.99%)	3393 (34.25%)	
*Physical activity (%)*				< 0.001
Low	4271 (36.83%)	804 (47.60%)	3467 (34.99%)	
Middle	2223 (19.17%)	295 (17.47%)	1928 (19.46%)	
High	5103 (44.00%)	590 (34.93%)	4513 (45.55%)	
Current smoking (%)	4082 (35.20%)	541 (32.03%)	3541 (35.74%)	0.003
Current drinking (%)	2606 (22.47%)	335 (19.83%)	2271 (22.92%)	0.005
Height (cm)	160.62 ± 8.21	157.03 ± 8.81	161.23 ± 7.95	< 0.001
Weight (kg)	64.13 ± 11.38	66.47 ± 12.38	63.73 ± 11.15	< 0.001
BMI (kg/m^2^)	24.80 ± 3.67	26.88 ± 4.17	24.45 ± 3.45	< 0.001
WC (cm)	82.43 ± 9.83	87.07 ± 10.00	81.64 ± 9.58	< 0.001
SBP (mmHg)	141.76 ± 23.44	160.01 ± 25.86	138.65 ± 21.51	< 0.001
DBP (mmHg)	82.05 ± 11.76	88.08 ± 13.73	81.02 ± 11.06	< 0.001
MBP (mmHg)	105.94 ± 15.19	116.85 ± 16.72	104.07 ± 14.09	< 0.001
Scr (μmol/L)	71.10 (63.00–79.80)	72.50 (63.40–82.20)	70.60 (63.00–79.60)	< 0.001
FPG (mmol/L)	5.55 (5.16–6.04)	5.72 (5.28–6.36)	5.52 (5.15–6.00)	< 0.001
TC (mmol/L)	5.23 ± 1.09	5.48 ± 1.19	5.19 ± 1.06	< 0.001
TG (mmol/L)	1.24 (0.88–1.89)	1.51 (1.04–2.27)	1.21 (0.86–1.82)	< 0.001
HDL-C (mmol/L)	1.41 ± 0.38	1.36 ± 0.36	1.42 ± 0.38	< 0.001
LDL-C (mmol/L)	2.93 ± 0.82	3.11 ± 0.91	2.89 ± 0.80	< 0.001
Anti-hypertensive therapy (%)	1753 (15.12%)	603 (35.70%)	1150 (11.61%)	< 0.001
Anti-diabetic therapy (%)	458 (3.95%)	117 (6.93%)	341 (3.44%)	< 0.001
Lipid-lowering therapy (%)	380 (3.28%)	114 (6.75%)	266 (2.68%)	< 0.001
Hypertension (%)	5925 (51.09%)	1381 (81.76%)	4544 (45.86%)	< 0.001
Diabetes (%)	1202 (10.36%)	301 (17.82%)	901 (9.09%)	< 0.001
CVD history (%)	2487 (21.45%)	596 (35.29%)	1891 (19.09%)	< 0.001
IVST (cm)	0.90 (0.80–0.90)	1.00 (0.90–1.10)	0.80 (0.80–0.90)	< 0.001
LVEDd (cm)	4.70 ± 0.46	5.04 ± 0.57	4.64 ± 0.41	< 0.001
PWT (cm)	0.80 (0.80–0.90)	1.00 (0.90–1.10)	0.80 (0.80–0.90)	< 0.001
RWT (cm)	0.36 (0.33–0.39)	0.39 (0.36–0.43)	0.36 (0.33–0.38)	< 0.001
LVM (g)	132.32 (113.63–158.21)	187.54 (162.94–220.26)	126.69 (109.69–146.83)	< 0.001
LVMI (g/m^2.7^)	36.98 (31.98–43.40)	53.91 (50.23–61.05)	35.43 (31.14–40.12)	< 0.001
ePWV (m/s)	11.25 ± 2.20	13.00 ± 2.15	10.95 ± 2.07	< 0.001

Data are summarized as mean (SD), median (interquartile range), and numbers (percentage) according to their data type and distribution

*LVH* left ventricular hypertrophy, *CNY* Chinese currency, *WC* waist circumstance, *BMI* body mass index, *SBP* systolic blood pressure, *DBP* diastolic blood pressure, *MBP* mean blood pressure, *FPG* fasting plasma glucose, *Scr* serum creatinine, *TC* total cholesterol, *TG* triglyceride, *LDL-C* low-density lipoprotein cholesterol, *HDL-C* high-density lipoprotein cholesterol, *IVST* interventricular septum thickness, *LVEDd* left ventricular end-diastolic diameter, *PWT* posterior wall thickness, *LVM* left ventricular mass, *LVMI* left ventricular mass index, *ePWV* estimated pulse wave velocity

*Chi-square test or Rank-sum test were employed to compare categorical variables between groups. Student's t test or Mann–Whitney test were employed to compare continuous data between groups

Logistic regression was employed to explore the association between ePWV and LVH prevalence and the results were displayed in Table 2. In the crude model, each SD increase of ePWV associated with a 2.700 times risk of LVH prevalence. After adjustment of age, sex, education, income, physical activity level, current smoking and drinking status, the risk for LVH prevalence for each SD increase of ePWV augmented to 3.500 times. With further adjustment of BMI, WC, Scr, TC, HDL-c, MBP, FPG, anti-hypertensive therapy, anti-diabetic therapy, lipid-lowering therapy, and CVD history, the OR for per SD increase of ePWV diminished to 2.993. When dividing ePWV into quartiles, the top quartile had a 4.520 times risk of LVH prevalence compared with the bottom quartile after adjusting for all the co-variates. Moreover, the results of P for trend test showed a linear trend of the association between ePWV quartiles and the LVH prevalence. To improve the clinical applicability of our results, we also performed logistic regression with RWT as outcome, the results were summarized in Additional file 1: Table S2. In the fully adjusted model, each SD increase of ePWV associated with a β value of 0.018 (95% CI 0.007–0.029, *p* = 0.002), and the top quartile had a β value of 0.040 (95% CI 0.012–0.069, *p* = 0.006) when compared with the bottom quartile.

**Table 2:** Multivariate logistic regression of ePWV for LVH prevalence

Variables	Odds ratio (95% CI)					
	Crude	*p* Value	Model 1	*p* Value	Model 2	*p* Value
ePWV (Per 1 SD increase)	2.700 (2.541, 2.868)	< 0.001	3.550 (3.228, 3.904)	< 0.001	2.993 (2.470, 3.628)	< 0.001
*Quartiles of ePWV*						
Quartile 1	Reference		Reference		Reference	
Quartile 2	2.346 (1.825, 3.016)	< 0.001	2.541 (1.967, 3.284)	< 0.001	1.524 (1.152, 2.018)	0.003
Quartile 3	5.874 (4.667, 7.393)	< 0.001	6.703 (5.216, 8.614)	< 0.001	2.700 (1.977, 3.686)	< 0.001
Quartile 4	14.222 (11.392, 17.755)	< 0.001	17.291 (13.150, 22.736)	< 0.001	4.520 (3.051, 6.698)	< 0.001
P for trend		< 0.001		< 0.001		< 0.001

Crude: no adjustment; Model 1: adjusted for sex, age, income, education, and physical activity level, current smoking and drinking status; Model 2: further adjusted for BMI, WC, Scr, TC, HDL-c, FPG, MBP, anti-hypertensive therapy, anti-diabetic therapy, lipid-lowering therapy, and CVD history

*ePWV* estimated pulse wave velocity, *LVH* left ventricular hypertrophy, *CI* confidence interval, *BMI* body mass index, *WC* waist circumference, *FPG* fasting plasma glucose, *MBP* mean blood pressure, *Scr* serum creatinine, *TC* total cholesterol, *HDL-C* high-density lipoprotein cholesterol, *CVD* cardiovascular disease, *SD* standard deviation

**Fig. 2:**
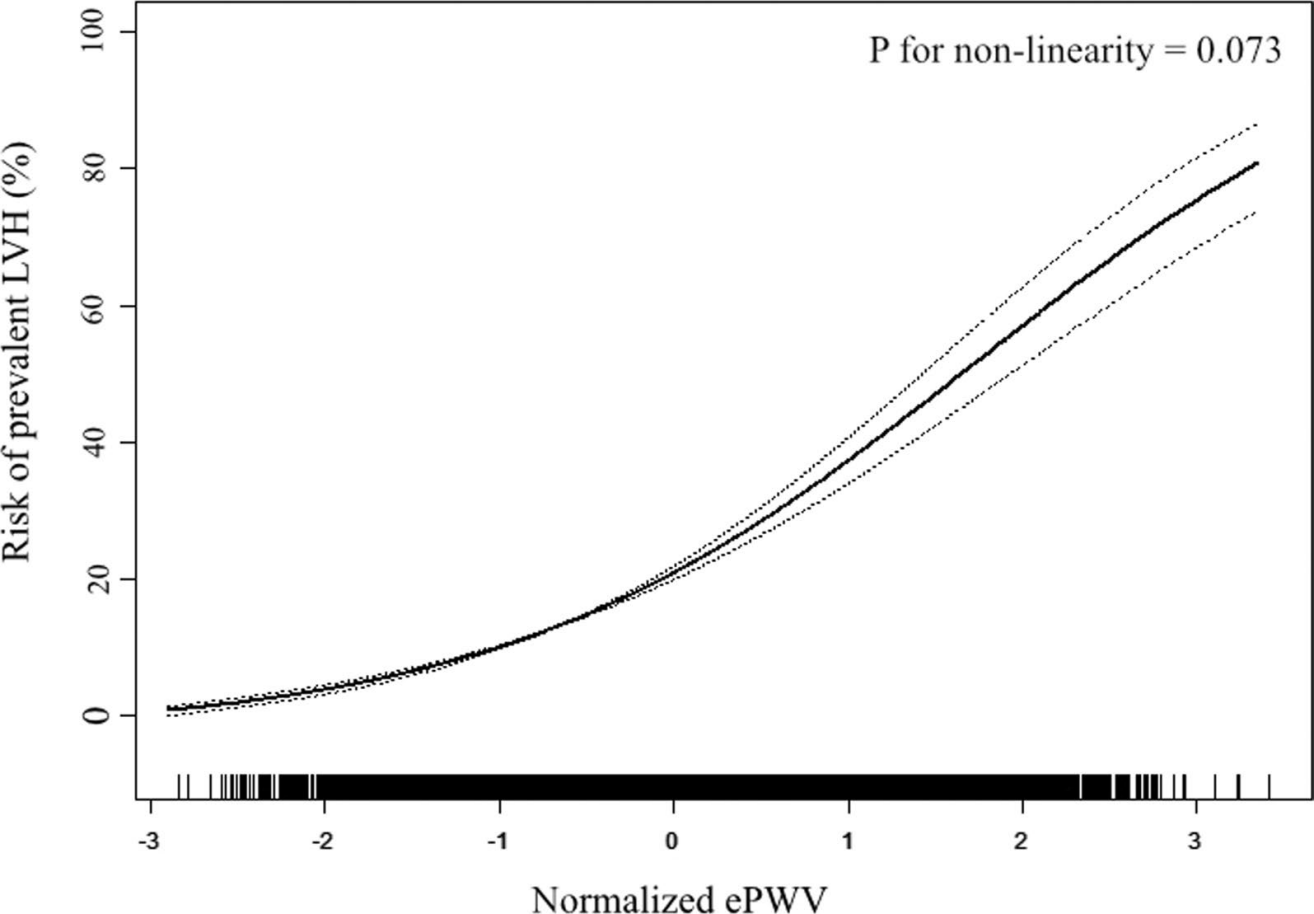
Smooth spline analysis of the association between ePWV and the risk of the presence of LVH. Smooth spline analysis was conducted through generalized addictive model with the adjustment of Clinical risk factors: age, sex, education level, income level, physical activity, current smoking, current drinking, BMI, WC, Scr, TC, HDL-c, FPG, MBP, anti-hypertensive therapy, anti-diabetic therapy, lipid-lowering therapy, and CVD history. In the plot, the risk of LVH prevalence increased proportionally with the increment of ePWV, and P for non-linearity was insignificant, suggesting the association between ePWV and LVH prevalence was linear in the whole range of ePWV

To investigate whether the association between ePWV and LVH prevalence was linear, our study employed a smooth curve fitting (Fig. 2). The plot showed a nearly linear association between normalized ePWV and LVH prevalence after adjustment of all co-variates used in the Model 2 of Table 2. Furthermore, *p* for non-linear association which based on logarithmic likelihood ratio test confirmed the association was linear in the whole range of ePWV (*p* = 0.073).

**Fig. 3:**
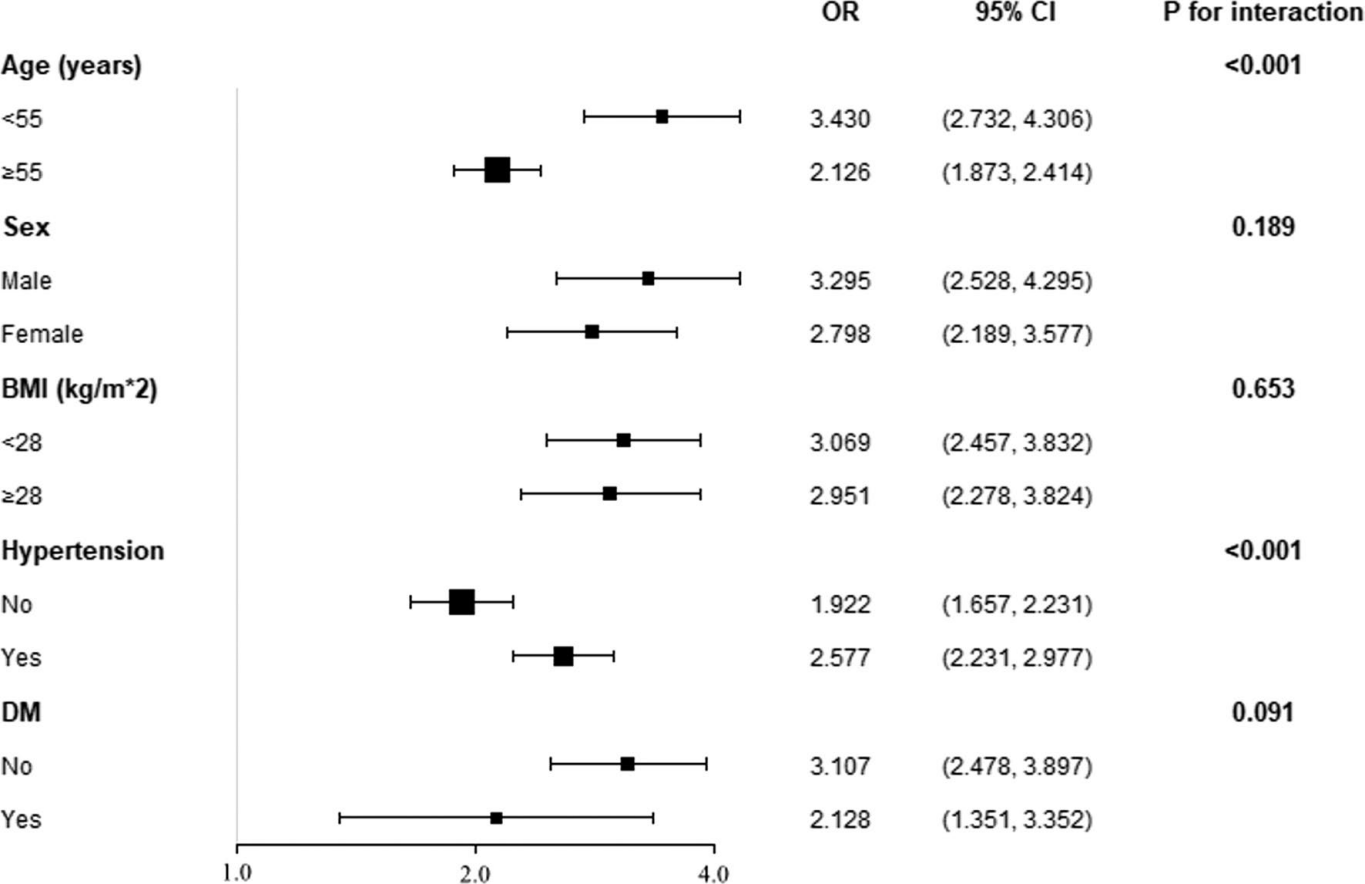
Subgroup analysis of the association between ePWV and LVH prevalence. The model in each stratum was adjusted for age, sex, education level, income level, physical activity, current smoking, current drinking, BMI, WC, Scr, TC, HDL-c, FPG, MBP, anti-hypertensive therapy, anti-diabetic therapy, lipid-lowering therapy, and CVD history except for the variate that was used to define subgroups (in HTN subgroups, MBP and anti-hypertensive therapy were not adjusted; in DM subgroups, FPG and anti-diabetic therapy were not adjusted). Subgroups of sex, obesity and diabetes showed insignificant interaction with the association between ePWV and LVH prevalence (*p* for interaction > 0.05). Significant interaction existed between age, hypertension, and the association between ePWV and the LVH prevalence. Younger people and hypertensive population were more vulnerable to the increase of ePWV than their corresponding counterparts regarding the risk of LVH prevalence

Subgroup analysis with interaction test was conducted to evaluate whether our major finding was robust in some common sub-populations (Fig. 3). For every stratum, the logistic regression model was adjusted for all co-variates used in Model 2 of Table 2, except for the variate that was employed for stratification (in HTN subgroups, MBP and anti-hypertensive therapy were not adjusted; in DM subgroups, FPG and anti-diabetic therapy were not adjusted). The results showed our major finding was robust in sub-populations of sex, BMI and diabetes. However, the association between ePWV and LVH prevalence was significantly different between subjects aged less than 55 and subjects aged equal to or more than 55 (*p* for interaction < 0.001). The younger sub-population had a significantly higher OR for LVH prevalence than the elder sub-population (3.430 vs. 2.126). In the hypertensive stratum, similar phenomenon was also observed, participants with hypertension had an OR for LVH prevalence of 2.577 (95% CI 2.231–2.977), significantly higher than that in participants with normal blood pressure (1.922, 95% CI 1.657–2.231).

Finally, our work conducted ROC analysis and reclassification analysis to identify the incremental value of ePWV to optimize the identification of LVH prevalence (Table 3). The AUC of ePWV for LVH prevalence was 0.758 (95% CI 0.750–0.765), significantly larger than that of MBP (0.725, 95% CI 0.716–0.733, *p* for comparison < 0.001). When adding ePWV into cardiovascular risk factors which included age, sex, education level, income level, physical activity, current smoking, current drinking, BMI, WC, Scr, TC, HDL-c, FPG, MBP, anti-hypertensive therapy, anti-diabetic therapy, lipid-lowering therapy, and CVD history, we observed a significant improvement for the identification of LVH prevalence (0.787 vs. 0.810, *p* for comparison < 0.001). In the reclassification analysis, NRI (0.421, 95% CI 0.370–0.471) and IDI (0.023, 95% CI 0.019–0.027) showed significant improvement for the classification of the presence of LVH or not when adding ePWV into cardiovascular risk factors. The reclassification table was presented in Additional file 1: Table S3.

**Table 3:** ROC and reclassification analysis for ePWV to improve the identification of LVH prevalence

Model	AUC (95% CI)	*p* Value	*p* for comparison	NRI (category free)	*p* Value	IDI	*p* Value
ePWV	0.758 (0.750, 0.765)	< 0.001	< 0.001	–	–	–	–
MBP	0.725 (0.716,0.733)	< 0.001		–	–	–	–
Clinical risk factors*	0.787 (0.780, 0.795)	< 0.001	< 0.001	–	–	–	–
Clinical risk factors + ePWV	0.810 (0.802, 0.817)	< 0.001		0.421 (0.370, 0.471)	< 0.001	0.023 (0.019, 0.027)	< 0.001

Clinical risk factors: age, sex, education level, income level, physical activity, current smoking, current drinking, BMI, WC, Scr, TC, HDL-c, FPG, MBP, anti-hypertensive therapy, anti-diabetic therapy, lipid-lowering therapy, and CVD history

*ROC* receiver operating characteristic curve, *LVH* left ventricular hypertrophy, *AUC* area under the curve, *CI* confidence interval, *NRI* net reclassification improvement, *IDI* integrated discrimination index, *BMI* body mass index, *WC* waist circumference, *FPG* fasting plasma glucose, *Scr* serum creatinine, *TC* total cholesterol, *HDL-C* high-density lipoprotein cholesterol, *MBP* mean blood pressure, *CVD* cardiovascular disease

## Discussion

In the current analysis, our data demonstrated a significant and positive association between ePWV and the LVH prevalence based on a general Chinese population. Furthermore, our results showed the association between ePWV and LVH prevalence was linear in the whole range of ePWV. Additionally, our findings suggested our major findings were robust in some common sub-populations, suggesting the value of ePWV in estimating LVH prevalence is applicable to these specified subgroups. Moreover, subjects aged less than 55 years old and participants with hypertension were more vulnerable to the increase of ePWV than their counterparts. Therefore, these sub-populations may deserve more monitoring of LVH and clinical care in the daily practice. Finally, our data showed a significant advancement in the identification of LVH prevalence when introducing ePWV into common cardiovascular risk factors. In general, our work suggests the potential value of ePWV, a simple surrogate of aortic stiffness, as a risk indicator to optimize the identification of LVH prevalence in the primary care settings.

Left ventricle is the target organ of multiple cardiovascular risk factors, especially hypertension and diabetes. And the most common result of the persistent damage to left ventricle is LVH. Published studies have identified high prevalence of LVH in hypertensive patients [6]. On the contrary, Successful lowering of the blood pressure into normal range has been identified to have a significant impact on LVH regression [26, 27]. Similar phenomenon was also observed among the diabetic patients. Prevalence of LVH in diabetic patients was identified to be higher than the general population [4, 28], and latest randomized control test had demonstrated the value of glucose-lowering drugs for the regression of LVH in diabetic patients [29], the plausible mechanism for this regression may be weight loss, improved insulin sensitivity, reduced blood pressure and ventricular load caused by glucose-lowering drugs. Meanwhile, aortic stiffness is another product of the persistent injury from multiple cardiovascular risk factors. The dominant risk factors for aortic stiffness were also hypertension and diabetes [30–33]. Accordingly, LVH and aortic stiffness may share similar pathophysiological etiology. Hence, scientists began to investigate the association between aortic stiffness and LVH, and the results showed significant association between markers of aortic stiffness and LVH [11–13]. However, the quantification of all the markers of aortic stiffness used in previous studies require expensive and specialized equipment, which is rarely equipped in the primary care condition. Therefore, there is an urgent need for a simple, rapid, and cost-effective surrogate to assess the severity of aortic stiffness. The newly proposed ePWV meets the demand, previous research had demonstrated the accuracy of ePWV in assessing the severity of aortic stiffness [16]. According to the above information, we hypothesized that ePWV is associated with the LVH prevalence and may improve the identification of LVH prevalence in the general population.

Findings from our data verified our hypothesis. After adjusting for demographic, anthropometric, laboratory, and medical history related co-variates, logistic regression revealed a significant and positive association between ePWV and LVH prevalence. The results showed the influence of ePWV on the prevalence of LVH, implicating the potential value of ePWV to act as a risk indicator of the presence of LVH. Additionally, the results from smooth spline analysis displayed that the association between ePWV and LVH prevalence was linear in the whole range of ePWV. Therefore, the risk of LVH prevalence may increase proportionally with the increment of ePWV, suggesting the potential of ePWV as a linear indicator to estimate the risk of LVH prevalence. Hence, it may be easy to use in the primary care settings.

To evaluate whether the significant association between ePWV and LVH prevalence was robust in some common subpopulations of cardiovascular diseases, our work conducted subgroup analysis based on age, sex, obesity (subjects were divided into 2 groups according to the obesity criteria of Chinese population [34]), hypertension, and diabetes. The results showed that there is no interaction between sex, obesity and diabetes and the association between ePWV and LVH prevalence, implicating that our major findings were robust to sex, obesity and diabetes, and the impact of ePWV on LVH prevalence was consistent in these subpopulations. However, in age and hypertension strata, there was a significant interaction between the stratifying variate and the association between ePWV and LVH prevalence. In the age strata, subjects aged less than 55 years old had a higher OR toward LVH prevalence than subjects aged equal to or more than 55 years old (3.430 vs. 2.126, *p* for interaction < 0.001), suggesting the younger population was more vulnerable to the increase of ePWV and the underlying aortic stiffness. In the hypertension strata, participants with hypertension had a significantly higher OR value than participants with normal blood pressure (2.577 vs. 1.922, *p* for interaction < 0.001), implicating that hypertension and aortic stiffness may act synergistically towards the development and progression of LVH. In general, the results of subgroup analysis suggest that the association between ePWV and LVH prevalence was consistent in sex, obesity, and diabetes strata, and for younger population and hypertensive patients, the increase of ePWV may cast more risk for LVH prevalence than their corresponding counterpart, therefore these subpopulations may deserve more attention and monitoring than the general population to prevent LVH development and progression.

It is necessary to mention that physical activity level could be associated with LVH in some population. In the current analysis, physical activity was documented and classified according to subjects’ answer towards our questionnaire. According to the definition of physical activity in our survey, even in subjects with high physical activity, few people would reach the activity level as professional athletes. Therefore, we believe the possibility that subjects with high physical activity and LVH present with a normal, physiological response to regular exercise is low. To confirm our hypothesis, we also conducted a cross-table for physical activity and LV geometry (Additional file 1: Table S2), the results showed high physical activity cast marginal influence on the LV geometry.

There is also a need to assess whether ePWV can detect the impact of lowering blood pressure on LVH regression. Among the 1753 subjects who received anti-hypertensive therapy, 603 (34.40%) had LVH, higher than that in the total population (14.56%) and that in all HTN patients (23.31%). It is worthy to mention that the HTN control rate among our HTN patients was only 5.97% (n = 354), even if 1753 (29.59%) of the HTN patients were using anti-hypertensive therapy. Among those with controlled HTN, the prevalence of LVH was 20.62% (n = 73), lower than that in all hypertensive patients but still higher than that in the total population. We employed logistic regression to assess the association between ePWV and prevalence of LVH in subjects with controlled HTN. However, due to the low rate of controlled HTN, the logistic regression in this subgroup was extremely lack of statistical power. Based on above data, we believe our results cannot answer whether ePWV can detect the impact of lowering blood pressure on LVH regression, further studies focusing on this topic are needed.

Our study further employed ROC and reclassification analysis to investigate the value of ePWV to optimize the identification of LVH prevalence in the general population. Although significantly better than MBP alone, ePWV itself still had limited value to identify LVH prevalence. Nevertheless, a significant improvement for the identification of LVH prevalence was observed when adding ePWV into cardiovascular risk factors (0.787 vs. 0.810, *p* for comparison < 0.001). However, as the most widespread method to investigate a new marker, ROC analysis still has its drawbacks. Published article has revealed the insensitivity of ROC analysis to detect the value of a new marker for improving the identification of diseases [35]. ROC analysis can compare the identifying ability of two models, but AUC cannot give an appropriate answer about whether adding a new marker into existed risk factors can optimize the accuracy of disease identification [36]. Hence, depending on ROC analysis alone may underestimate the value of a new marker. Based on above information, statisticians have proposed reclassification analysis (including IDI and NRI) to assess the incremental impact of adding a new marker to established risk factors for disease identification [37–39]. As both NRI and IDI were significant, our findings from the reclassification analysis support the significant incremental value of ePWV to optimize the identification of LVH prevalence. Accordingly, both ROC analysis and reclassification analysis suggest the usefulness of ePWV to improve the identification of people with high risk of LVH prevalence. Through introducing ePWV into primary care settings, clinicians would have more objective evidence to achieve early identification of LVH prevalence and then make appropriate clinical decisions. In general, our findings implicate the incremental value of ePWV to improve the identification of LVH prevalence in the general population.

Except from sharing a similar profile of risk factors, aortic stiffness itself can also cause LVH. Mechanical fatigue and fragmentation of elastin fibers cause dilatation of the aorta. Therefore, the pressure load is transferred to the stiffer elements of the aortic wall, such as collagen. The consequence of this pathophysiological change is the increment of aortic wall stiffness and pulse wave velocity, and then results in that the reflected pressure waves will arrive at late systolic phase rather than diastolic phase [40]. Hence, the systolic pressure will increase and thereby augment the afterload of left ventricle [41, 42]. Eventually, long-term persistent increase of the afterload of left ventricle will lead to LVH.

Our current analysis still has limitations. Firstly, our current work only used cross-sectional data of the survey, follow-up data was unavailable because the follow-up work was delayed during the COVID-19 pandemic. Therefore, the data of our work can only suggest the association between ePWV and LVH prevalence, the longitudinal association between ePWV and the development and progression of LVH still needs prospective studies to explore. Secondly, our subjects were sampled from the natural population in the southeastern China. Hence, our findings from the current data may not be applicable for people from different areas or countries with diverse race and socioeconomic conditions. Thirdly and lastly, as the same with other observational research, unrecorded variates can also cause residual confounding and thereby bring bias into our analysis. For example, the echocardiography in our survey could only provide cardiac chamber parameters. Therefore, we could not add valvular heart diseases such as aortic stenosis into our analysis. To address this disadvantage, more studies with more detailed information collection are needed to verify the association between ePWV and the LVH prevalence. Lastly, although NRI and IDI are novel and interesting statistical methods for investigating the capacity of new markers to improve the prediction and identification of outcomes, they still have limitations. They are both composite scores that include reclassification of risk in both an upwards and a downwards direction. Therefore, they cannot provide information about the specific direction of reclassification, and they have relatively high false positive rate to detect the significant value of new markers. Furthermore, although IDI was significant in our current work, it was low. Hence, more studies evaluating the association between ePWV and LVH prevalence in different populations are needed in future.

## Conclusion

In summary, our current analysis showed a positive, linear association between ePWV, a simple surrogate of aortic stiffness, and the risk of LVH prevalence in a general Chinese population. Furthermore, our data demonstrated that the association was robust in the sex, obesity and diabetes subpopulations, and younger people and hypertensive population were more vulnerable to the increase of ePWV than their corresponding counterparts. Moreover, our findings revealed the incremental value of ePWV to optimize the identification of LVH prevalence in the general population, implicating ePWV may serve as a simple and cost-effective marker to improve the early identification and prevention of LVH.

## Supplementary Information

**Additional file 1. Table S1.** Cross-table evaluating the association between physical activity and LVH prevalence; **Table S2.** Multivariate linear regression assessing the association between ePWV and RWT; **Table S3.** Reclassification table for identifying LVH prevalence by models with and without ePWV.

## Data Availability

Data were available from the corresponding author (Xu Liu) in request if appropriate.
