# Supplementary material for: Value of estimated pulse wave velocity to identify left ventricular hypertrophy prevalence: insights from a general population
Source: BMC Cardiovasc Disord. 2022 Apr 8;22:157. doi: 10.1186/s12872-022-02541-9 (PMC8990685; doi:10.1186/s12872-022-02541-9)
Supplement: Supplementary file 1 — Additional file 1. Table S1. Cross-table evaluating the association between physical activity and LVH prevalence; Table S2. Multivariate linear regression assessing the association between ePWV and RWT; Table S3. Reclassification table for identifying LVH prevalence by models with and without ePWV. [file 12872_2022_2541_MOESM1_ESM.docx]

Supplemental materials

Table S1. Cross-table evaluating the association between physical activity and LVH prevalence.

| LV geometry | normal geometry | concentric remodeling | eccentric hypertrophy | concentric hypertrophy |
| --- | --- | --- | --- | --- |
| Physical activity |  |  |  |  |
| low (%) | 2991 (72.28) | 343 (8.29) | 565 (13.65) | 239 (5.78) |
| middle (%) | 1728 (79.85) | 141 (6.52) | 201 (9.29) | 94 (4.34) |
| high (%) | 4078 (81.94) | 309 (6.21) | 419 (8.42) | 171 (3.44) |
| Overall (%) | 8797 (78.00) | 793 (7.00) | 1185 (10.50) | 504 (4.50) |

Abbreviations: LVH: left ventricular hypertrophy; LV left ventricle.

Table S2. Multivariate linear regression assessing the association between ePWV and RWT.

| Variables | β (95% CI) | | | | | |
| --- | --- | --- | --- | --- | --- | --- |
|  | Crude | P value | Model 1 | P value | Model 2 | P value |
| ePWV (Per 1 SD increase) | 0.013 (0.007, 0.019) | <0.001 | 0.022 (0.011, 0.032) | <0.001 | 0.018 (0.007, 0.029) | 0.002 |
| Quartiles of ePWV |  |  |  |  |  |  |
| Quartile 1 | Reference |  | Reference |  | Reference |  |
| Quartile 2 | 0.012 (-0.005, 0.030) | 0.167 | 0.017 (-0.001, 0.035) | 0.070 | 0.014 (-0.005, 0.033) | 0.149 |
| Quartile 3 | 0.012 (-0.005, 0.029) | 0.173 | 0.021 (-0.000, 0.042) | 0.053 | 0.015 (-0.007, 0.037) | 0.185 |
| Quartile 4 | 0.036 (0.019, 0.054) | <0.001 | 0.050 (0.024, 0.076) | <0.001 | 0.040 (0.012, 0.069) | 0.006 |
| P for trend |  |  |  |  |  |  |

Crude: no adjustment; Model 1: adjusted for sex, age, income, education, and physical activity level, current smoking and drinking status; Model 2: further adjusted for BMI, WC, Scr, TC, HDL, hypertension, diabetes, cardiovascular disease history.

Abbreviations: ePWV: estimated pulse wave velocity; RWT: relative wall thickness; CI: confidence interval; BMI: body mass index; WC: waist circumference; FPG: fasting plasma glucose; MBP: mean blood pressure; Scr: serum creatinine; TC: total cholesterol; HDL-C: high-density lipoprotein cholesterol; SD: standard deviation.

Table S3. Reclassification table for identifying LVH prevalence by models with and without ePWV.

|  | New Model | | | Reclassified (%) |
| --- | --- | --- | --- | --- |
|  | 0-3% | 3-10% | >10% |  |
| Old Model | No. | No. | No. |  |
|  |  | Group 2 (n=9908) |  |  |
| 0-3% | 1093 | 128 | 5 | 11 |
| 3-10% | 805 | 3330 | 442 | 27 |
| >10% | 1 | 716 | 3388 | 17 |
|  |  | Group 1 (n=1689) |  |  |
| 0-3% | 13 | 5 | 1 | 32 |
| 3-10% | 15 | 184 | 82 | 35 |
| >10% | 0 | 49 | 1340 | 4 |

Old model: Included age, sex, education level, income level, physical activity, current smoking, current drinking, BMI, WC, Scr, TC, HDL-c, FPG, MBP, anti-hypertensive therapy, anti-diabetic therapy, lipid-lowering therapy, and CVD history.

New model: Old model + ePWV.

Abbreviations: ePWV: estimated pulse wave velocity; LVH: left ventricular hypertrophy; BMI: body mass index; WC: waist circumference; FPG: fasting plasma glucose; MBP: mean blood pressure; Scr: serum creatinine; TC: total cholesterol; HDL-C: high-density lipoprotein cholesterol; CVD: cardiovascular disease.
